# Unlink the Link Between COVID-19 and 5G Networks: An NLP and SNA Based Approach

**DOI:** 10.1109/ACCESS.2020.3039168

**Published:** 2020-11-18

**Authors:** Mohammed Bahja, Ghazanfar Ali Safdar

**Affiliations:** 1 College of Engineering and Physical Sciences (EPS)University of Birmingham 1724 Birmingham B15 2TT U.K.; 2 School of Computer Science and TechnologyUniversity of Bedfordshire 5195 Luton LU1 3JU U.K.

**Keywords:** 5G conspiracy, corona-5G link, COVID-19, radiation scare, topic modelling, tweet analysis

## Abstract

Social media facilitates rapid dissemination of information for both factual and fictional information. The spread of non-scientific information through social media platforms such as Twitter has potential to cause damaging consequences. Situations such as the COVID-19 pandemic provides a favourable environment for misinformation to thrive. The upcoming 5G technology is one of the recent victims of misinformation and fake news and has been plagued with misinformation about the effects of its radiation. During the COVID-19 pandemic, conspiracy theories linking the cause of the pandemic to 5G technology have resonated with a section of people leading to outcomes such as destructive attacks on 5G towers. The analysis of the social network data can help to understand the nature of the information being spread and identify the commonly occurring themes in the information. The natural language processing (NLP) and the statistical analysis of the social network data can empower policymakers to understand the misinformation being spread and develop targeted strategies to counter the misinformation. In this paper, NLP based analysis of tweets linking COVID-19 to 5G is presented. NLP models including Latent Dirichlet allocation (LDA), sentiment analysis (SA) and social network analysis (SNA) were applied for the analysis of the tweets and identification of topics. An understanding of the topic frequencies, the inter-relationships between topics and geographical occurrence of the tweets allows identifying agencies and patterns in the spread of misinformation and equips policymakers with knowledge to devise counter-strategies.

## I. Introduction

Information can be a boon, when it is reliable, authorised, and validated. On the contrary, it can be a curse if it is misused or overloaded. Information is a key resource in handling serious issues such as pandemics which requires effective dissemination of quality and reliable information across all stakeholders including public. However, increase in the use of social media technologies such as Facebook, WhatsApp, YouTube etc., have empowered individuals in disseminating information reflecting their personal thoughts and perceptions about an issue, sometimes even in spreading hatred or myths or conspiracies. A small dissemination of misinformation on online platforms can lead to serious issues which not only affects people but also the governments and the society as a whole [1].

The recent incidents of attacks on 5G towers/masts signify the importance of understanding the nature and attitudes of the public in sharing misinformation and the process of how myths related to 5G were developed and rapidly spread. The inconsistencies between the trusted and the non-trusted sources have to be investigated and reasoned in order to prevent the spread of misinformation and change peoples’ attitudes towards unauthorised information sources and wrong information. By adopting innovative technologies like Artificial Intelligence (AI), Machine Learning (ML), and NLP, the online information, their sources and the pattern and process of sharing of the information can be analysed. A recent study by Groza [2] used Description Logics for detecting inconsistencies between trusted medical sources and non-trusted ones. The study has identified that non-trusted information comes in natural language, while trusted information comes in a more formal language. Therefore, applying semantic reasoning and NLP techniques can identify the relationships between the types of information and how they were shared by the public.

The work presented in this paper focuses on investigating the factors that led to violent attacks on 5G infrastructure by reviewing and analysing the tweets related to 5G and COVID-19 in the UK using NLP techniques and text-mining techniques. Investigating these factors may not only contribute to the information management and awareness creation during COVID-19 pandemic, but also can be used to develop strategies to prevent information misuse in future in similar situations.

Statistical analysis of the tweets can unearth vital information related to the geographical spread of misinformation, and the frequently occurring terms and themes in the misinformation. This knowledge can be used to develop customized approaches to counter misinformation. For instance, geographic location of the tweets and its occurrence frequency (discussed in V-D) can identify the hotspots or regions that are more prone to consumption of misinformation. Granular data about the nature of the information from a geographical information can help local governmental agencies to create strategic awareness programs to counter the spread of misinformation and potentially reduce the further spread of fake news and conspiracy theories. The objective of the paper is to apply NLP techniques on the tweet dataset related to COVID-19 and 5G to perform statistical analysis and identify themes and topics from the tweets to understand the spread of misinformation.

The rest of the paper is organised as follows. Section II discusses the recent events of spread of misinformation related to the COVID-19 pandemic and its link to the 5G technology, i.e. *the Myth*. Section III presents some well-known relevant models adopted in our experiments and subsequent outcomes/analysis. The experiments conducted are presented in Section IV, the results and analysis are outlined in Section V. Finally, Section VI discusses our work and the limitations of our study, followed by conclusion in Section VII.

## II. The Myth

Various myths and misinformation have been circulating on online platforms in relation to the recent COVID-19 outbreak, which have resulted in severe losses. For instance, rumours such as drinking raw alcohol as a cure for COVID-19 in Iran has resulted in many deaths [3]; similarly, conspiracy theory linking 5G with COVID-19, has resulted in more than 20 attacks on masts in the UK [4]. In this context, Singh *et al.* [5] identified that a meaningful spatio-temporal relationship exists between myths and are linked to poor quality information on Twitter discussions. Therefore, there is an immediate need for containing the spread of misinformation on online platforms and increased public awareness through various channels by using an evidence-based approach.

Jelnov [6], worked on delinking the myth and reported that the virus is not very dangerous by correlating the log of tests and reported cases, as well as the reported cases and deaths per capita. Their work suggested mortality rate of 0.4% from COVID-19 in a cross-country comparison. However, Constantinou *et al.* [7] argued that science has been failing to convince people about COVID-19 findings and suggested that measures need to be taken. They identified that myths and conspiracy theories were believed even by highly educated individuals and that such beliefs could be predictors of health-related risky behaviour, such as refusing social distancing, pushing for mass gatherings for demonstrations, and refusing future vaccinations.

In a different context, Laato *et al.* [8] investigated why people share misinformation during COVID-19 Pandemic, and revealed that a person’s trust in online information and perceived information overload to be strong predictors of unverified information sharing. In addition, these factors, along with a person’s perceived COVID-19 severity and vulnerability influence cyberchondria [8]. Similarly, in a study conducted by Allington and Dhavan [9], a strong acceptance was exhibited by the public (in the UK) in relation to the conspiracy belief that ’the symptoms of COVID-19 seem to be connected to 5G mobile network radiation’, in contrast to other conspiracy beliefs such as ’the virus was created in a lab’ and ’COVID-19 pandemic was planned by pharmaceutical companies’. Similarly, Cushion *et al.* identified that the UK public were more involved in identifying and circulating fake news, rather than identifying the important information about UK death toll and the impact of COVID-19 on the UK population [10].

## III. NLP Models

### A. LDA Models

Latent Dirichlet Allocation (LDA) [11] is a generative model of topic modelling widely used in the literature and has shown good performance in analysing large, noisy datasets [12]. The LDA method is an unsupervised approach and can identify themes and topics from a dataset without requiring the dataset to be annotated [13]. The approach followed by LDA assumes that each topic is a distribution of words and each document has a certain distribution of topics. Variations of LDA modelling are identified based on the number of words used to define a topic and are referred as n-gram modelling [14]. For instance, unigram models identify topics from distribution of single words and bigram models identify topics from a distribution of pair of words. LDA models enable identification of topics in the document thereby generating observations and in turn a group of observations can be associated to identify recurring topics in the document.

### B. Sentiment Analysis

Sentiment analysis (SA) is a natural language processing method to identify the sentiment or opinion contained in a given piece of data [15]. As opinion is subjective, sentiment analysis extracts the *subjectivity* in the given text [16]. SA classifies the opinion or the identified subjectivity in the text into different classes, most frequently, into binary classifications, such as positive sentiment or negative sentiment. SA enables a computational study of people‘s opinion, sentiment, attitude, and emotion towards an entity. The entity can be about another individual or a public figure, a product, such as cinema or electronic device or service providers, such as restaurants and hospitals. Recent advancements in machine learning is well explored for SA and several studies are available based on techniques such as support vector model [17], [18], naives bayes [19], [20], strength of association [21], and advanced deep learning approaches [22]–[24]. SA is explored for applications such as user reviews [25], feedback forum analysis [26], patient experience [27], social media data analysis [28], market intelligence [29], public mood observations, and similar applications.

### C. Social Network Analysis

Social network analysis (SNA) approach provides methods to determine relationships between entities (e.g., people or groups) [30]. In our study, SNA is performed using centrality-based social network method for network analysis [31] and on co-occurrence analysis [32] of words to visualize the network. The co-occurrence analysis identifies the frequency of keywords that belong to similar themes and topics and describes the relationship among the keywords. Further, the co-occurrence network for noun bi-grams is constructed to visualize the relationships between the different terms in the network.

## IV. Experiments

### A. Dataset Collection and Pre-Processing

The data presented by [33] is used in our experiments. The dataset is the first publicly available coronavirus related multi-lingual twitter dataset. The tweets were collected from 28 January 2020 onwards using application programming interface (API) provided by twitter. With various COVID-19 pandemic related keywords, tweets from as early as 21 January, 2020 were recognized in the dataset. Over 50 million COVID-19 related tweets are indexed by the dataset. For more details on the dataset, please refer to [34].

The focus of our work requires COVID-19 tweets in the context of 5G technology, therefore the pre-processing step involved filtering out the tweets. The keywords presented in Table 1 were used to filter out COVID-19 tweets pertaining to 5G. The tweets identified are dated from 21 January, 2020 until 18 April, 2020. Further pre-processing operations included removal of duplicates by tweet ID and duplicated contents. Post filtering and removal of duplicates, a total of 82,043 tweets were available for analysis. Other standard data cleaning steps mentioned in Table 2 were applied during the pre-processing stage.

**TABLE 1:** Keywords to Filter the Tweets Corpus

Keywords
fifth generation
Wireless communications
Towers radiation
Radiation poisoning
5G radiation

**TABLE 2:** Data Cleaning Prior to Analysis

Pre-processing step	Function
Fix abbreviations	Replace short words with full words
Remove irrelevant characters	Remove redundant characters including links, email IDs
Fix word lengthening	Removal of additional, repeated characters
Stopword removal	Removal of words including “the”, “an” and customized stopwords
SpellCheck	Fix wrong spelling
Punctuation removal	Remove punctuations
Lemmatization	Group words with similar meanings to a single item

### B. Optimal Topic Number

The first stage of our analysis concerned finding the optimal number of topics that represents the contents of the evaluated dataset and focused on topics consisting of nouns only. Multiple LDA models with a topic number $N$ ranging from $N=2$ to $N=38$ were evaluated based on coherence scores as a metric. The coherence score assesses the quality of the learned topics by measuring the relative distances between the words in a topic [35]. A high coherence score of the topics indicate the high probability of the words belonging to a particular topic.

**FIGURE 1.**
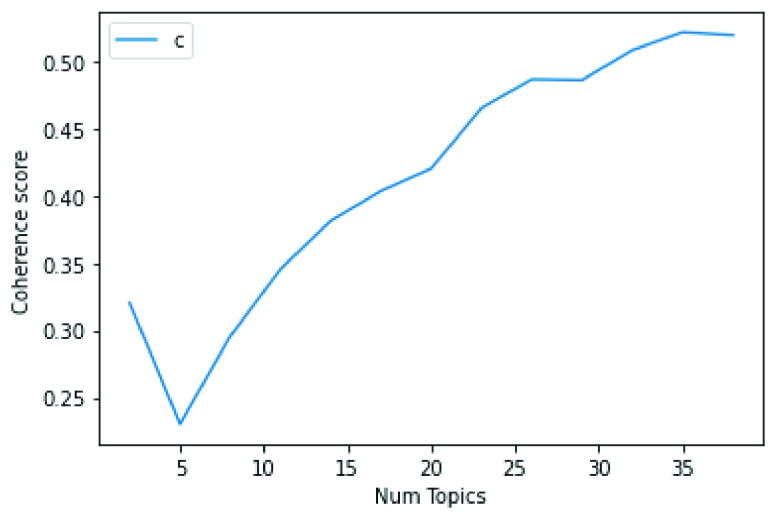
Coherence score for different number of topics.

From the evaluation of our LDA model, it was observed that the highest coherence score of 0.52 is obtained for the topic number, N=35. Therefore, the optimal number of topics to represent our dataset under evaluation is $N\_{}topic=35$. Figure 1 shows the coherence value score against the $N\_{}topic$ evaluation.

**FIGURE 2.**
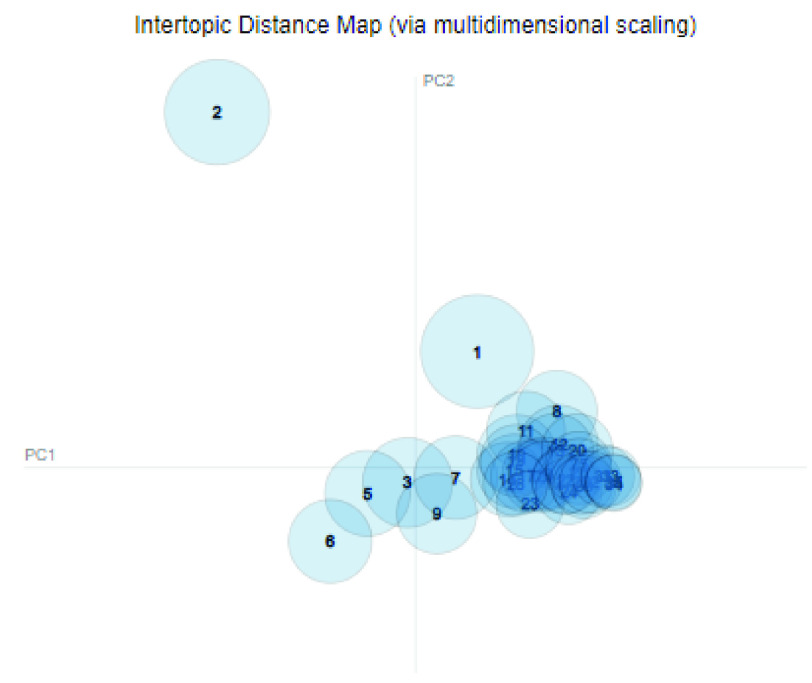
Intertopic distance map for N=35 number of topics.

Finally, after 150 iterations of LDA analysis at a $N\_{}topic=35$, the LDA model for topics with nouns only was built. A intertopic distance map presented in [36] allows to visualize the distance between the identified topics. The distance map is a reflection of how similar or distinct the topics are from each other and the relative size of the topics. Figure 2 visualizes the intertopic distance map of the 35 topics recognized by the LDA model.

## V. Results and Analysis

### A. LDA Analysis

The LDA analysis via unigram and bigram modelling was applied on the dataset under eight different study conditions. Each variation attempts to identify topics from full-text, nouns, adjective, verbs and adverbs. Despite the optimal number of topics was identified to be $N\_{}topic=35$, however, our LDA implementation was restricted to 20 topics because after approximately 20 topics the distribution of words tend to be repetitive and do not provide meaningful insights than identified. The eight LDA analysis studies identified 20 topics each and each topic represented by distribution of 20 words. Once the topics were identified in each LDA attempt, the word distribution in each topic were manually analyzed and labelled. Table 3 shows an example labelling of the word distribution of an identified topic.

**TABLE 3:** Manual Labelling of a Topic for a Word Distribution Identified by the LDA Model

Words belonging to a topic				Topic Label
coronavirus	pastor	covid	conspiracy	5G conspiracy
medium	chris	radiation	comment	
cause	video	china	world	
holmes	virus	technology	corona	
eamonn	news	effect	use	
china	huawei	network	security	5G threat
coronavirus	decision	technology	trump	
boris	trade	canada	johnson	
supply	deal	role	risk	
britain	threat	contract	government	

**FIGURE 3.**
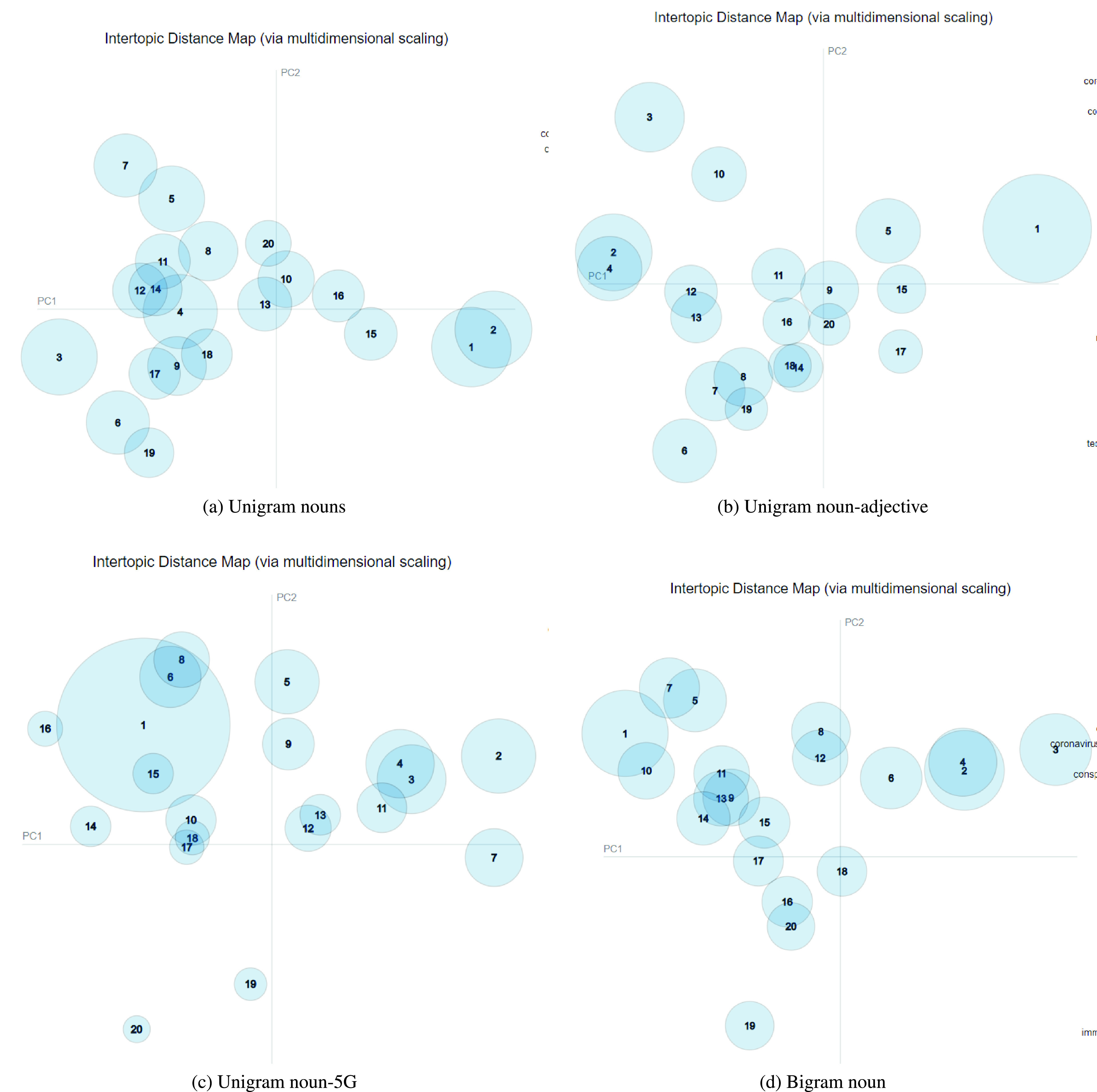
Intertopic distance map of topics identified of four LDA analysis studies: (a) Unigram Nouns (b) Unigram Noun-adjective pair (c) Unigram noun-5G words (d) Bigram nouns.

The entire analysis comprising of eight topics pertaining to LDA model is presented in Table 4, inclusive of: *unigram model; unigram nouns; unigram nouns and adjectives; unigram nouns, adjectives, verbs and adverbs; unigram nouns and 5G keywords; bigram plain text; bigram nouns;* and *bigram nouns and adjectives*. It can be observed from the Table 4 that several topics are repeated and are closely related. The reason for similar and repeated topics is the homogeneity of the dataset under evaluation. Topic modelling shows better performance with diverse dataset that contain heterogeneous categories (e.g: news articles) with little to no restriction of target areas. In our study, we place a restriction of only 5G related COVID-19 tweets thereby restricting the heterogeneity of the dataset and leading to clustering and duplication of topics. For instance, Figure 3 shows the inter-topic distance map of four of the eight LDA analysis studies. The distribution of topics varies largely in each study, however, it can also be observed that the intertopic distance is relatively low and the topics tend to cluster. The inter-topic distance map is inspired by the *LDAvis* method of topic visualization presented by Sievert and Shirley [36].

**TABLE 4:** Topics Identified for Different Attempts

Topics Identified							
Uni-gram topics				Bi-gram topics			
Clean-text	Nouns	Nouns-adjectives	Noun-Adj Verb-Adverb	Noun-5G Keyword	Clean-text	Nouns	Nouns-adjectives
5G rollout	5G conspiracy	corona spread	coronavirus spread	5G radiation spread	5G conspiracy theory	conspiracy theory	dismiss conspiracy
5G tower	5G radiation	5G rollout	radiation effects	5G rollout threat	Huawei threat	radiation effects	5G spreads corona
5G network spread	Chinese companies threat	Huawei threat	5G tower	corona vaccination	5G conspiracy	huawei links	conspiracy theory
5G phone usage	5G secretive	lockdown causes	Chinese companies	5G effects	5G tower	war speculation	5g tower conspiracy
Chinese companies	China control	radiation effects	%G conspiracy	Huawei politics	radiation effects	huawei global threat	radiation global threat
Chinese product security	Debunk conspiracy	coronavirus effects	Radiation effects	Huawei threat	Huawei threat	corona diagnosis	huawei threat
5G causes	5G towers	5G tower	huawei threat	Huawei global dominance	5G threat	huawei threat	corona epidemic effects
5G Conspiracy	Diagnosis treatment	radiation effects world	technology during corona	technology during corona	5G conspiracy	5G tower	5g tower threats
5G radiation effects	Dismiss conspiracy	chinese mobile companies	5G conspiracy	corona spread	corona spread	radiation fears	conspiracy theory
5G links	5G causes	technologies during pandemic	5G conspiracy	Blame for virus	5G Conspiracy	huawei link	5G threat UK
Investigate 5G	5G towers	5G effects	5G rollout	5G effects health	5G threats	conspiracy theory	corona awareness
5G spread	Radiation effects	chinese companies	Tower effects	5G global threat	5G conspiracy	5G causes	5G links
5G causes	Radiation causes	5G conspiracy	huawei threat	5G tower	5G conspiracy theory	huawei global threat	conspiracy theory
5G radiation	5G threat	virus spread	corona effects	5G threat	Huawei links	corona news	discrimination corona
Chinese companies	Huawei threat	5G conspiracy	radiation effects	5G threat	conspiracy theory	5G links	huawei links
5G links	radiation effects	5G radiation	lockdown	war	huawei link	corona awareness	radiation threats
5G spread	tower conspiracy	5G conspiracy	corona effects	Huawei threat	corona spread	Huawei global threat	conspiracy theory
Chinese companies	Huawei threat	5G tower effects	Huawei threat	Technology effects	Huawei global risk	5G radiation	radiation threats
5G conspiracy	5G conspiracy	5G conspiracy	war	Huawei threat	conspiracy theory	corona news	china conspiracy
5G radiation effects	corona effects	Chinese companies	corona spread	conspiracy	5G links huawei	conspiracy theory	huawei threat

The intertopic distance map shown in V-A includes a graph with axes placed as principal component 1 (PC1) and principal component 2 (PC2) and correspond to the principal components of the topic space. In the context of the presented research, the x-axis can be seen as representing the subject of “coronavirus” and the y-axis represents the subject “5G”. Each quadrant can be interpreted to indicate the relevance of topics to different subjects. The topics in first quadrant implies that the tweets are related to both the topics of coronavirus and 5G. Similarly, on the second quadrant the topics are more related to 5G, the topics in third quadrant are not significantly related to neither of the topics, and topics in the fourth quadrant imply that the tweets are more related to coronavirus and less related to 5G. The topic centres are determined by computing the Jensen-Shanon divergence [37] between topics that measures the similarity between two probability distributions and with multi-dimensional scaling the inter-topic distances are projected onto two dimensions. The areas of the circle are proportional to the prevalence of the topics in the corpus.

### B. Sentiment Analysis

In the second part of our work, we performed sentiment analysis (SA) on the identified topics from the LDA models. SA on the LDA topics classifies whether the topic carries a positive or negative sentiment. Identifying the overall sentiment score of the topics provides insights into the ‘emotions’ carried by the topics. A negative trending score implies tweets carry negative emotions such as unhappiness, anger, fear, and others. The Valence Aware Dictionary and sentiment Reasoner (VADER) model of SA presented in [38] was implemented for identifying the sentimentality of the topics. VADER is a popular model of SA and has demonstrated good performance in various studies. In the second iteration of our study, the VADER model was applied on the topics identified. The approach of identifying the general sentiment of a specific topic helps in determining the general emotion behind the tweets. For all tweets $\sum _{i=1}^{n}tw_{i}$ of a topic $T_{i}$, a mean sentiment score $S_{T_{i}}$ is calculated by finding the sentiment score for all tweets belonging to the topic.

**FIGURE 4.**
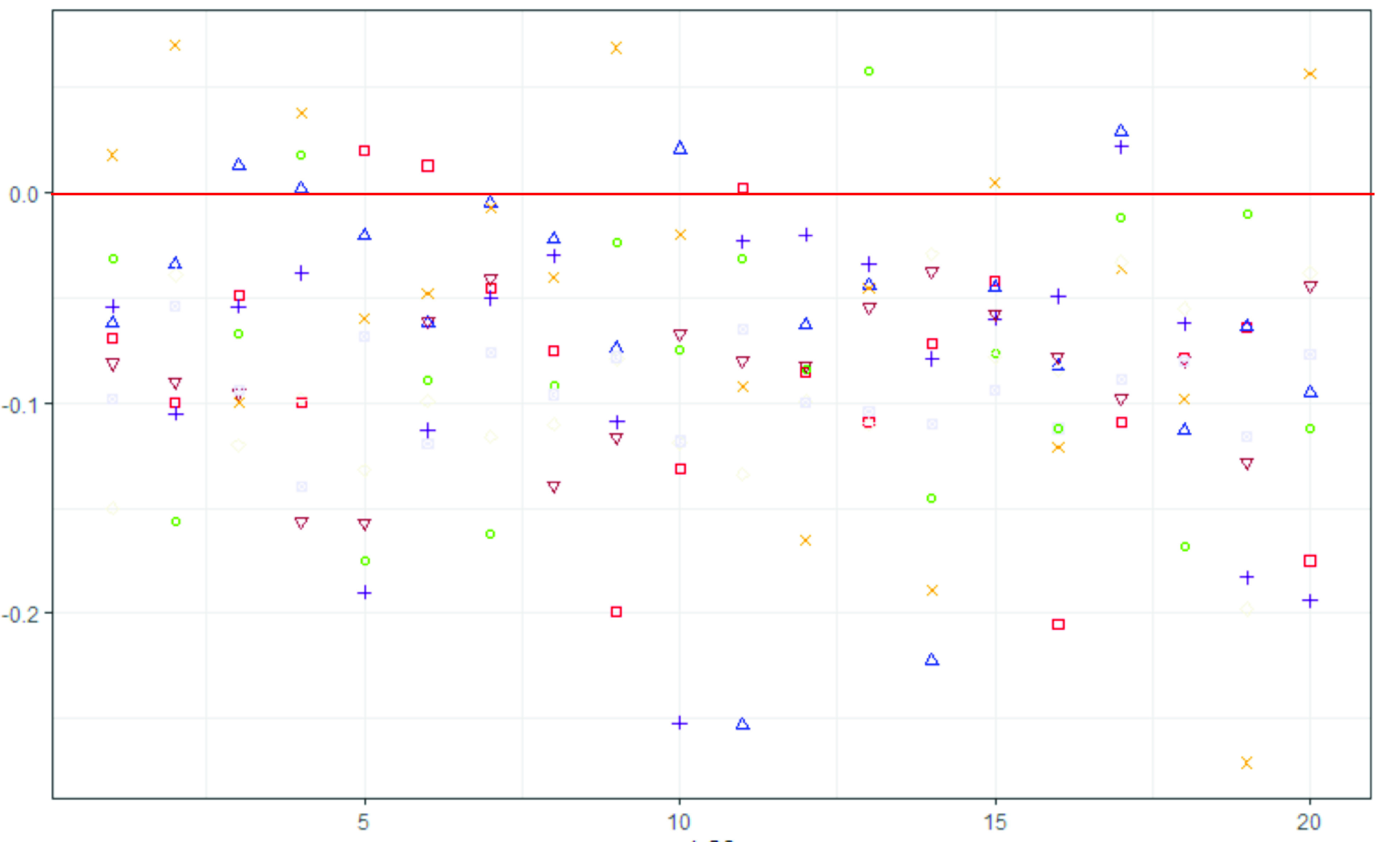
Sentiment score distribution of the topics identified. Positive sentiment topics are represented above the zero axis (red line).

Figure 4 illustrates the sentiment scores identified of all the topics belonging to our eight step analysis. The topics identified with positive sentiment scores have a value above zero and are plotted in the figure. The positive scored topics are highlighted with the green background. It can be observed that the majority of the topics belong to negative scores as shown in the graph indicating that the 5G related COVID-19 tweets have largely carried negative sentiment that may include emotions such as anger, hatred or fear.

### C. Social Network Analysis (SNA)

A co-occurrence network consists of nodes and edges. Nodes are objects or agents that are connected through the edges that defines the connection between the nodes. To identify co-occurring words, bigrams are identified from the tweets through nodes and edges. The identified bigrams describes the co-occurrence between them.

Prior to the network analysis, several pre-processing steps were performed on the dataset for the network construction. The pre-processing steps included filtering out tweets prior to January 2020, eliminating inconsistencies in the naming of the term “coronavirus”, removal of one-letter words, lemmatizing nouns, and other steps such as eliminating set entities, combining/trimming named entities, and space removal. The processed dataset is analyzed to identify the bigrams and build the co-occurrence network. Table 5 provides the statistics of the network analysis. It can be observed that the number of nodes is lower than the number of edges. The connection between the nodes (i.e. edges) leads to the identification of bigrams and thus $N$ unique bigrams are identified. In Table 6, the top 20 nodes and the bigrams identified from the nodes are listed.

**TABLE 5:** Descriptive Statistics of the Nodes co-Occurrence

	Measure	Statistic
0	N bigrams	205562
1	N unique bigrams	130381
2	Mean bigram frequency	1.57663
3	Number of nodes	19102
4	Number of edges	120014
5	Average degree	12.5656

**TABLE 6:** Top 20 Bigrams and Their Occurrence Frequency

	Bigram	Count
0	(cause, coronavirus)	1268
1	(conspiracy, theory)	1012
2	(coronavirus, conspiracy)	514
3	(coronavirus, coronavirus)	497
4	(coronavirus, cause)	268
5	(spread, coronavirus)	268
6	(link, coronavirus)	255
7	(china, huawei)	227
8	(immune, system)	223
9	(coronavirus, outbreak)	212
10	(theory, coronavirus)	204
11	(base, station)	179
12	(kill, people)	171
13	(people, coronavirus)	170
14	(people, cause)	170
15	(huawei, network)	157
16	(coronavirus, people)	141
17	(connection, coronavirus)	139
18	(coronavirus, virus)	134
19	(china, coronavirus)	132

Further analysis of the network is performed by determining the node importance in the network. The analysis in our study is centrality measures based and is applied to determine the influential keywords from the dataset. The centrality measures are determined via *degree centrality* and *betweenness centrality* [39]. The degree centrality of a node is the number of all edges of a node relative to the combined number of edges normalized by dividing by the maximum possible degree in a simple graph n-1. The degree centrality indicates the importance or the influence of a particular node on the network. The betweenness centrality is defined as the fraction of all possible shortest paths between any pair of nodes that pass through the node, i.e. the more frequently a node acts as a “bridge” to connect other nodes to each other, the higher the centrality. Table 7 shows the degree centrality and betweenness centrality of the top-20 nodes.

**TABLE 7:** Top 20 Nodes With Centrality Measures

	Betweenness centrality		Degree centrality	
	Node	Betweenness	Node	Degree centrality
0	coronavirus	0.209812	coronavirus	0.198995
1	china	0.126235	china	0.149940
2	people	0.043556	people	0.082771
3	network	0.036148	network	0.071096
4	virus	0.031834	virus	0.068164
5	cause	0.031083	cause	0.059526
6	huawei	0.026467	huawei	0.059159
7	technology	0.026042	technology	0.056437
8	world	0.024576	world	0.055966
9	use	0.021242	use	0.050835
10	conspiracy	0.018418	conspiracy	0.046071
11	country	0.017610	country	0.045600
12	radiation	0.016119	time	0.043191
13	time	0.015268	radiation	0.041935
14	phone	0.013227	government	0.037380
15	news	0.012584	tower	0.035757
16	vaccine	0.012084	phone	0.035653
17	tower	0.011107	system	0.034763
18	government	0.010763	vaccine	0.033558
19	system	0.010098	thing	0.032773

Figure 6 shows the network visualization of different nodes within the network. The nodes are connected through the edges (i.e. lines). The thicker the lines, the stronger is the relationship between the two nodes. The network reflects the bigram-noun model. The nodes in the diagram are similar to the words identified in the noun-bigram topic modelling version. It can be noted that the node “coronavirus” has the highest number of edges in the graph and has stronger relationship with nodes such as “telecom”.

**FIGURE 5.**
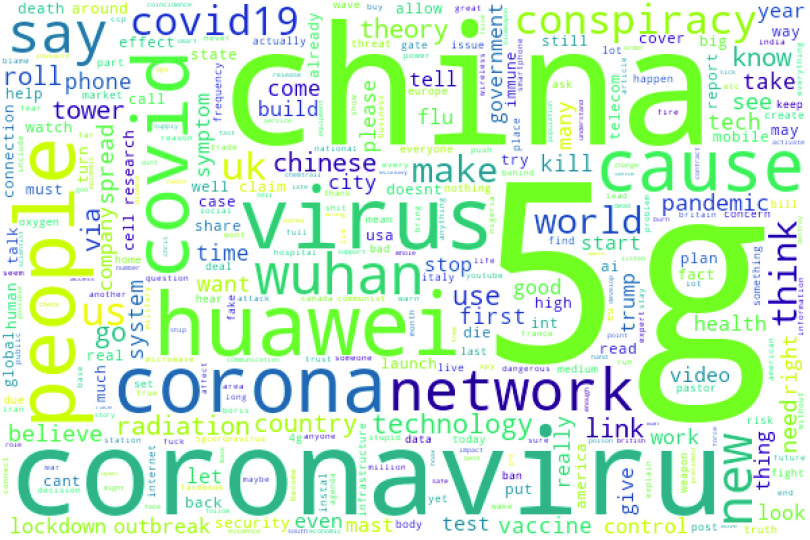
WordCloud of the frequently occurring terms.

**FIGURE 6.**
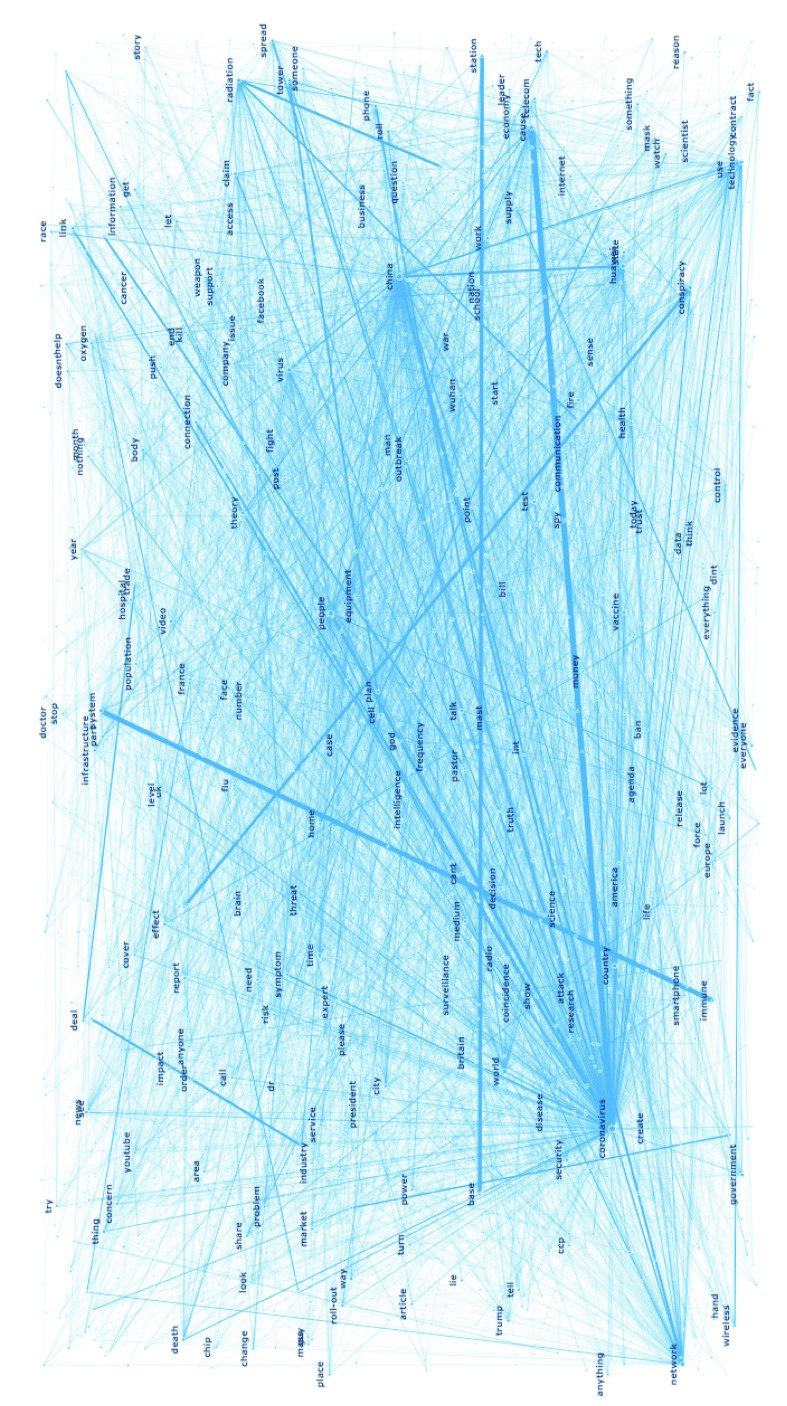
Co-occurrence network indicating the relationship between the nodes and the strength between each of the nodes.

### D. Statistical Analysis

The most commonly occurring words on 5G related COVID-19 tweets were analyzed from the dataset and the LDA model outcomes.

#### 1) Tweet Frequency – Geographical Occurrence

The occurrence of 5G related COVID-19 tweets according to countries was analyzed. Table 8 lists the top countries with the highest number of tweets on 5G-COVID related subject. It can be noted that highest amount of tweets were observed in USA, UK, Canada. The geographical occurrence of the tweets is significant as it correlates to the spread of misinformation and damaging consequences. For instances, in the UK where a high occurrence of tweets happened a significant number of cases of attacks on 5G masts were reported [40], [41]. The amount of tweets, geolocation of tweets and the speed of spread can be a vital tool for agencies to counter misinformation and focus to create awareness at target areas.

**TABLE 8:** Top Three Countries With Highest Number of 5G-COVID19 Tweets

Country	Number of tweets
USA	~ 7500 tweets
UK	~ 5000 tweets
Canada	~ 2000 tweets

#### 2) Word Frequency - Dataset

The first analysis identified the most frequently occurring words in the dataset. Wordcloud analysis was applied to identify the most frequent words. Figure 5 is a WordCloud visualization of the most frequent words. It can be observed that apart from the obvious ‘5G’ and ‘coronavirus’ words, the most frequent words include ‘China’, ‘Huawei’, ‘network’, ‘technology’, ‘radiation’, ‘tower’, etc.

#### 3) Word Frequency – LDA Models

**FIGURE 7.**
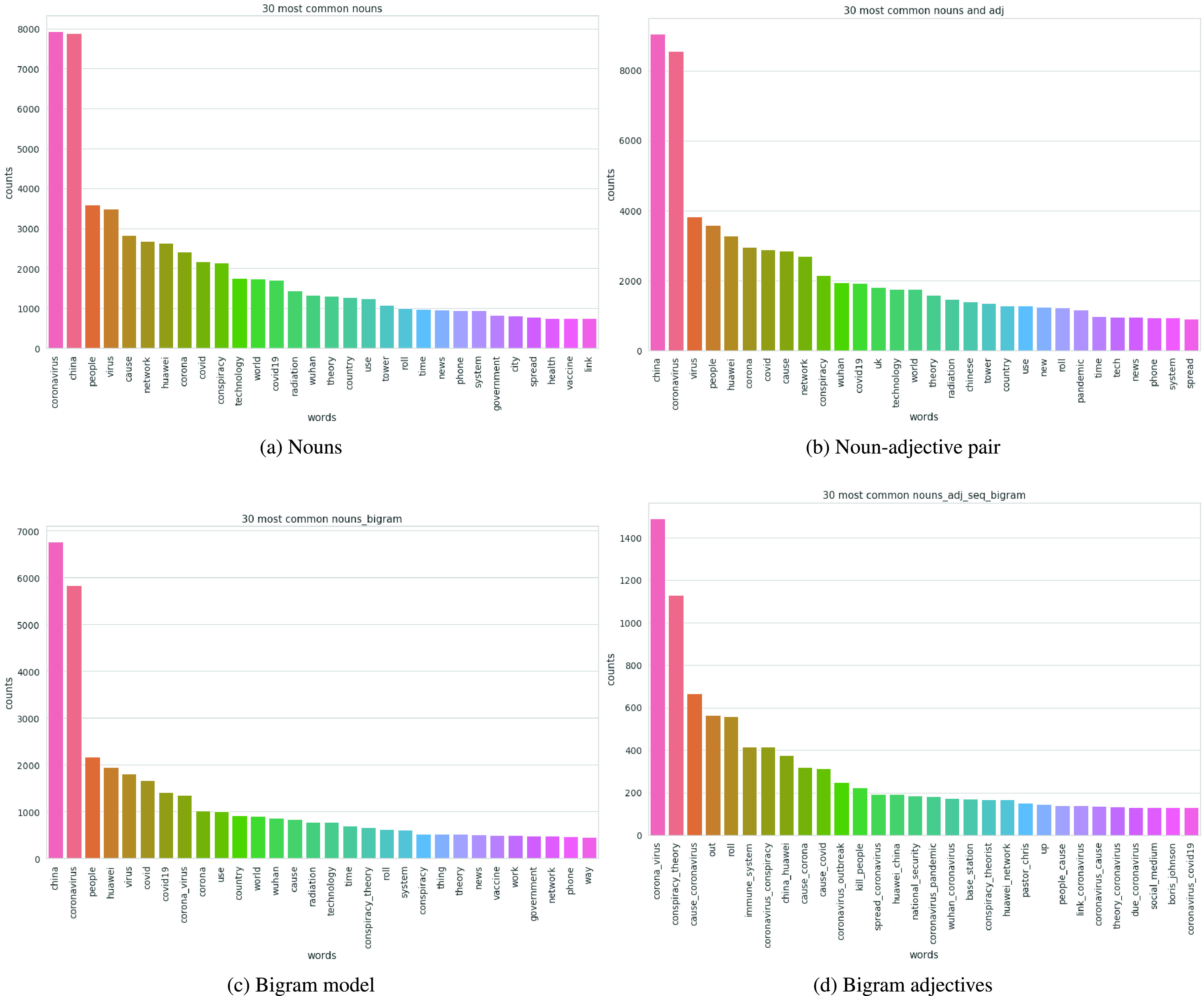
Frequently occurring terms identified during LDA analysis for different attempts: (a) Noun (b) Noun-adjective pair (c) Bigram word (d) Bigram adjectives.

Similarly, word frequency analysis were performed on the topics identified in each of the LDA model. In Figure 7, word frequency count for four out of the eight LDA model variations evaluated is displayed. It can be noted again that, apart from the 5G and COVID-19 related terms, some of the most frequently occurring words are 5G technology related words such as ‘Huawei’, ‘network’, ‘technology’, ‘radiation’, ‘conspiracy’.

The most frequent words indicate that the technology related words are frequently propagated in the tweets. Further, occurrence of terms like ‘conspiracy’ and frequent nouns such as ‘China’, ‘Huawei’, ‘Trump’, ‘USA’ indicate the manifestation of political clashes into conspiracies and spread of misinformation.

## VI. Discussion - Limitation of Study

A pandemic situation provides a conducive environment for spread of false information. Non-scientific claims such as 5G radiation effects found significant boost during the COVID19 crisis. Social media platforms such as Twitter provide miscreants an effective tool to accelerate the rate of misinformation spread. In this paper, we attempted to analyse the tweets related to 5G and COVID19 scare with a goal to understand the tweet trends. The LDA analysis of the tweets enables us to identify several topics from the tweets. An overview of the topics identified indicates that the majority of the topics speak about the conspiracy behind the COVID19 pandemic and is evidenced by large corpus of tweets that believe that the 5G technology causes COVID19.

Our analysis observed that China and Huawei were frequently discussed in the tweets. Similarly, frequently occurring terms and discussed topics include 5G towers, radiation effects, network and radiation. The majority of topics are related to 5G radiation and tower effects and conspiracy theories against China and Huawei.

Our study satisfies the evaluation metrics proposed by Camache *et al.*’s four dimensions of social network analysis [42]. The evaluation metrics are – (a) *Pattern and knowledge discovery*: our study identifies the themes and topics from the tweet corpus; (b) *Scalability*: the presented approaches can work on larger datasets and potentially allow application of deep learning based techniques; (c) *Information fusion and integration*: text data from different social media platforms can be included for further analysis and is our potential future work; (d) *Visualization*: the *LDAvis* approach and the other data visualizations presented in our study provides an insightful representation of the themes and topics of the tweet data.

It is believed that an understanding of the themes and trends from the tweets is crucial for policymakers to counter the misinformation with correct targeted information. Further, identifying the geographical location of the tweets and themes of the tweet propagated in the region can be a useful information for agencies to design awareness programs specific to the target area and the population.

The results presented in the study has limitations mainly due to the homogenous data used in the analysis. As the tweets are narrowed down to the 5G and COVID19 topic, the LDA analysis identifies themes that are clustered and overlap. However, the analysis provides vital information about the recurring themes across the tweets. Further, sentiment analysis tools provide additional information about the overall emotion associated with the topics.

One of the limitation of the study is the relatively lower number of tweets available for analysis. The smaller corpus is due to the restriction of the tweets to just the topic of COVID and 5G. A larger number of tweet corpus can provide more robust and insightful analysis.

The study can be enhanced with the application of machine learning and deep learning techniques for further analysis. Techniques such as word2vec models [43] are to be explored for more detailed analysis. Due to the homogeneity of the dataset focusing on 5G, applying deep learning based advanced approaches might not give robust results. Inclusion of other conspiracy topics apart from 5G into the dataset can help in improving the variety and veracity of the dataset to apply deep learning based NLP methods.

## VII. Conclusion

NLP based analysis of social media data provides opportunities to understand the nature and spread of misinformation. The COVID-19 tweets linking the pandemic to 5G were analysed to identify the recurring themes and topics within the tweets. Models including LDA, sentiment analysis and social network analysis were applied for the analysis of the tweets and identification of topics. An understanding of the topic frequencies, the inter-relationships between topics and geographical occurrence of the tweets enables to detect agencies and patterns in the spread of misinformation and equips with policymakers with knowledge to devise counter-strategies. The research work certainly can benefit and improve further by focusing on more granular analysis of the data and longitudinal analysis of the nature of information spread.
